# Urethral Calculi in the Dog1Read before the meeting of the Pennsylvania State Veterinary Association, March 7, 1900.

**Published:** 1900-04

**Authors:** Howard B. Felton

**Affiliations:** Philadelphia, Pa.


### URETHRAL CALCULI IN THE DOG.[1]

By Howard B. Felton, B.S., V.M.D.,
PHILADELPHIA, PA.

It is not the purpose of this paper to cover the whole field of urinary calculi in the dog, but simply to speak of the occurrence and method for treatment of those calculi most commonly found, viz., urethral calculi. Calculus of the urinary organs is very

[1] Read before the meeting of the Pennsylvania State Veterinary Association, March 7, 1900.

common in the dog, and probably many cases go unsuspected because the calculi formed are very small and pass through the natural channels without ever forming an obstruction. The healthy urine of the dog differs from that of vegetable-feeders in the absence of hippuric acid, in its clear consistency, bright yellow color, and strong odor. In subjects affected with calculus it becomes more or less cloudy, and deposits a gritty sediment on standing. In some cases it is red, and contains blood corpuscles; in others it is yellow and thick, as if containing pus, but really contains urine crystals.

The calculi found in the urethra of the dog,and we speak here of the male, although they are found at rare intervals in the female, are in most cases small, rounded, or spherical in shape, and varying in size from a mustard-seed to that of a large pea. Occasionally they are triangular shaped, or very irregular in outline, and with a rough, harsh surface which grates under the sound. As a rule, the smooth stones may be said to be formed of carbonates, phosphates, and oxalates of lime and magnesia, and of silica, while the rough, jagged crystalline specimens consist of ammonia-magnesian phosphates. Uric acid is found in the composition of many of these calculi either as free acid or in the form of urates.

In regard to the causes for the formation of calculi we find urea, uric acid, creatin, creatinin, leucin, tyrosin, etc., are all nitrogenous elements formed from the waste of muscular and gelatinous tissues or from albuminoid matters in the food. Urea is the healthy product of such decomposition, while uric and hippuric acids are products in which the oxidation has stopped short, leaving the products in a less soluble condition and more liable to crystallize out. According to Professor Law, impaired breathing, from diseased lungs or otherwise, and imperfect action of the liver, whether from local diseases in that organ or from feverish states with impaired functions generally, are therefore among the causes which strongly predispose to urinary calculi. A certain amount of mucus, fat, coloring matter, and blood enter into the formation of these concretions. Anything which favors the concentration of the urine may be mentioned as an accessory cause. As in the formation of watery vapor in the air it is necessary to have a solid nucleus for it to condense upon, so in the formation of calculi we find the first requisite is that some solid body should exist as a nucleus, around which layer after layer is crystallized, and hence we find these stones are built up in a series of concentric layers. The nucleus may consist of a particle of mucus, fibrin, or blood, a crystal deposited from oversaturated urine, or even a foreign body introduced from without. Professor Law mentions having seen a large calculus in the kidney of a deer, formed around a piece of wood which must have penetrated the kidney and broken off, while the wound by which it entered had healed up. Boerhäve introduced a small stone into the bladder of a dog, and it became the nucleus of a large concretion.

It is very common to find an impaction of a number of small round stones cemented together by mucus into a spherical mass and lodge just above the penial bone. This form of impaction may give the impression of a single large calculus. The impaction may extend up the urethra to the ischio-pubic arch, or in rare cases to the neck of the bladder. The small calculi have a remarkable property of embedding themselves in the walls of the urethra, which makes it very difficult to dislodge them.

We have noticed that this trouble is much more common in the small breed of dogs, and have found it to be most frequent in skye-terriers than in any other breed.

Urethral calculi are supposed by most authors to be formed in the bladder. This is doubtless the source of the larger calculi; but we are inclined to the opinion, formed as the result of several post-mortems, that the smaller calculi have their origin in the ureters or in the uriniferous tubules.

The diagnosis of urethral calculus is comparatively easy. Usually the patient will be brought to you with the statement that he has difficulty in passing his urine. You will notice that the back is more or less arched, the end of the penis usually protruding from the sheath, and if allowed to run around the dog will generally make frequent efforts to urinate, but will only be able to eject a few drops with difficulty and sometimes with great pain, and there will be more or less dropping of the urine from the end of the penis after the effort to expel it has ceased. Examination of the course of the urethra externally will disclose the fact that it is distended above the penial bone. It is only necessary to pass the sound to complete the diagnosis, when the obstruction can be plainly felt, usually immediately above the penial bone. We believe it to be good practice to pass the catheter in any suspected trouble of the urethra or bladder, as we have known practitioners to overlook both urethral calculus and enlarged prostate from a neglect of this precaution. In some cases we all find the obstruction to the flow of urine complete, and these cases must be quickly relieved before uræmic poisoning and mortification set in. Some cases can be relieved by judiciously using a probe of suitable calibre to break up the obstructing mass, when it will be carried away by the flow of urine aided by the expulsive force of the bladder.

A client of ours, Mr. Charles Rees, a watchmaker, has devised a couple of very ingenious instruments for removing these calculi. He is the owner of a skye-terrier that we were obliged to operate on three times for this trouble. From the knowledge we were able to impart, and knowing the nature of the obstruction and its situation, he constructed two instruments. One is fashioned somewhat after a crochet-needle, with all the sharp edges that could wound the urethra filed down; this is designed to pass over the stone with the point and by careful manipulation force it into the cavity below the point, when it may be withdrawn; the handle is formed in the shape of a ring, the termination of which serves to indicate the location of the cavity below the point when it has been introduced into the urethra. The other instrument is designed to crush the stone or cut into its surface, reducing its calibre. Mr. Rees has used these instruments a number of times upon his dog, and has always succeeded in relieving him.

When all other means fail, or the calculus is too large to be removed with instruments introduced into the urethra, resort must be had to the knife. After locating the stone with the sound, cut boldly down upon it, making the incision in the median line of the urethra. Usually it is not necessary to make an incision more than one inch in length. When the stone is located high up in the urethra, and the subject is very small, it will sometimes tax all the patience and the resources of the operator to remove it. It has been our practice to leave the wound open if the incision is small, as there will generally be a number of small calculi passed for a day or two, coming down from above the obstruction, and the open wound facilitates their removal. Urine will be passed through the wound for three or four days, after which it will close and the dog will then urinate in the natural way.

Occasionally a case will be brought to you in an almost moribund condition, where the obstruction has existed so long that uræmic poisoning and commencing mortification of the bladder has set in. Operation under these circumstances we have found will not avail, and should not be attempted.

Preventive treatment—change of diet from animal to vegetable food, demulcent drinks, drinking of alkaline waters—has been recommended, but we have never found any benefit from their use, possibly because our instructions have not been fully carried out.

It would seem that the habit of the system which favors the formation of the calculi, once formed, persists, and remains to trouble the subject during his natural life.

